# Unraveling the Significance of Fecal MicroRNA Profile in Alzheimer’s Disease

**DOI:** 10.1007/s12035-025-05626-6

**Published:** 2025-12-22

**Authors:** Kavisha Katuwawala, Prashant Bharadwaj, Ian Martins, B. G. D. N. K. De Silva, Vincent Ho, Asiri Dissanayake, Ralph N. Martins, W. M. A. D Binosha Fernando

**Affiliations:** 1 Centre of Excellence for Alzheimer’s Disease Research & Care, School of Medical and Health Sciences, Edith Cowan University, Joondalup, WA 6027 Australia; 2 Alzheimer’s Research Australia, Ralph and Patricia Sarich Neuroscience Research Institute, Nedlands, WA 6009 Australia; 3 Department of Biomedical Sciences, Faculty of Medicine, Health and Human Sciences, Macquarie University, Sydney, NSW 2109 Australia; 4 Genetics and Molecular Biology Unit, Faculty of Applied Sciences, University of Sri Jayewardenepura, Nugegoda, 10250 Sri Lanka; 5 School of Medicine, Western Sydney University, Penrith, NSW 2751 Australia; 6 Prabhodha Hospitals Pvt Ltd., Ampara, 32000 Sri Lanka

**Keywords:** Alzheimer’s disease (AD), MicroRNAs (miRNAs), Gut–brain axis, Gut microbiota

## Abstract

Alzheimer’s disease (AD), a complex neurodegenerative disorder characterised by progressive cognitive decline, affects millions globally. With no current cure, treatment focuses on symptom management and slowing disease progression. Early identification is therefore crucial. Fecal microRNA (miRNA) analysis is emerging as a promising non-invasive diagnostic tool. Evidence suggests that miRNAs present in fecal samples may regulate gene expression linked to AD pathology, impacting cellular functions and disease progression. Dietary factors critically influence gut microbiota composition and diversity, affecting brain health via the gut brain axis. Macronutrients such as carbohydrates, proteins, and fats modulate miRNA expression and gut microbiota, influencing AD risk. High-fat diets can increase inflammation and contribute to AD. This review aims to provide a comprehensive overview of how diet-modulated fecal miRNAs and gut microbiota interplay may serve as novel, non-invasive indicators for early detection and intervention strategies in AD.

**Supplementary Information:**

The online version contains supplementary material available at 10.1007/s12035-025-05626-6.

## Introduction

AD is a progressive neurodegenerative disorder characterised by a gradual decline in cognitive abilities, including memory, language, reasoning, and motor function [1]. The pathology of AD is defined by two hallmark features: the accumulation of amyloid-beta (Aβ) plaques and the formation of hyperphosphorylated tau neurofibrillary tangles (NFTs). These abnormalities disrupt neural communication, trigger chronic neuroinflammation, and ultimately lead to synaptic dysfunction and neuronal death. However, to date, the precise mechanisms underlying AD onset and progression remain poorly understood [2]. Unlike blood and cerebrospinal fluid (CSF), which primarily reflect systemic and CNS-derived miRNA alterations, fecal miRNAs offer a gut-specific perspective that directly captures host–microbe interactions within the gastrointestinal tract. Circulating and CSF miRNAs such as miR-29a, miR-132, miR-107, and miR-186-3p reflect amyloid metabolism, synaptic dysfunction, and early cognitive decline [3–6], but they do not represent the local regulatory dynamics occurring in the gut. In contrast, faecal miRNAs including miR-146a, miR-155, miR-223, and miR-128 participate in shaping microbial communities such as *Bacteroides fragilis*, *Clostridium*, *Escherichia/Shigella*, *Enterobacteriaceae*, and SCFA-producing genera like *Faecalibacterium*, *Roseburia*, and *Eubacterium* [4, 7–11]. By integrating microbial composition, inflammatory responses, and metabolic signaling, fecal miRNAs provide additional mechanistic insight into gut–brain axis dysfunction not captured by blood or CSF markers. Thus, fecal miRNA profiling offers a non-invasive, complementary biomarker platform for investigating how gut pathology contributes to AD progression [4, 12].

In recent years, miRNAs, small non-coding RNAs that regulate gene expression, have emerged as important contributors to the pathophysiology of neurodegenerative diseases, including AD. miRNA studies have mainly used blood and tissue samples, but recently, stool samples have been considered as a new, less invasive option [13]. Stool samples offer unique advantages as they are easy to collect, allow for repeated sampling, and are particularly suitable for longitudinal studies [14]. The investigation of miRNAs in relation to AD stems from their potential role in the gut–brain axis, which is a bidirectional communication network between the gastrointestinal tract and the central nervous system (CNS). This axis plays an important role in neurodevelopment, immune regulation, and the pathogenesis of neurodegenerative disorders [15]. miRNAs found in stool can originate from both the host and gut microbiota, and emerging evidence suggests that microbiota-derived miRNAs may influence key processes involved in AD pathology (Aβ accumulation, tau hyperphosphorylation, neuroinflammation, and synaptic degradation) [16]. For example, microbial miRNAs such as miR-155 and miR-21 have been shown to play important roles in AD progression [17, 18]. However, findings are inconsistent across studies, largely due to differences in study design, sample handling, sequencing methods, and bioinformatic analysis. These methodological variations limit reproducibility and hinder clinical translation [16–19]. Multiple factors influence miRNA expression in both the host and gut microbes. These include age, diet (especially fibre and polyphenol intake), inflammation, immune status, and medications like antibiotics. Stress, sleep, metabolic health, genetics, sex, and environmental exposures also play roles. Additionally, sample handling and sequencing methods can affect results, emphasising the need for standardisation in miRNA studies. This review examines the current literature on the role of gut microbiota and microbial miRNAs in AD pathology, with a particular focus on their potential as biomarkers for early detection. We highlight the challenges in this emerging field, including inconsistent findings and methodological gaps, and propose directions for future research [20].

## Alzheimer’s Disease

AD is a progressive neurodegenerative disorder and the most common cause of dementia, currently impacting over 35 million people worldwide, a number projected to increase significantly with the ageing global population [21, 22]. The clinical features of AD typically involve a gradual decline in cognitive functions, particularly memory, accompanied by behavioural and psychological disturbances. Neuropathologically, the disease is characterised by extracellular accumulation of Aβ plaques, intracellular neurofibrillary tangles composed of hyperphosphorylated tau protein, synaptic degeneration, and chronic neuroinflammatory responses [23]. AD is a multifactorial condition arising from a complex interplay of genetic predispositions, environmental exposures, metabolic dysregulation, and modifiable lifestyle factors. Despite extensive research efforts, no disease-modifying therapy has yet been established, and current interventions remain limited to symptomatic relief [24, 25].

### Pathology of AD

Several hypotheses have been proposed to describe the pathology, including Aβ aggregation, tau protein hyperphosphorylation, mitochondrial dysfunction, and oxidative stress [26–28]. In addition to these, novel theories such as prion-like propagation of protein aggregates [29], cerebral vasoconstriction and vascular contributions to cognitive decline [30], and the role of altered gamma oscillations in network dysfunction [31] have also been proposed. Emerging evidence also points to the hormone secretagogue receptor 1α (GHSR1α) and its influence on synaptic plasticity and neurodegeneration [32] as well as chronic infections such as Herpes simplex virus type 1, as potential contributors to the development of AD [33]. The deposition of Aβ is a hallmark feature of AD and plays a central role in the pathogenesis and progression of the disease. Aβ is derived from a larger protein called amyloid precursor protein (APP). In a healthy brain, APP undergoes processing by α-secretase or β-secretase, producing soluble, non-toxic fragments that are efficiently metabolised or cleared, preventing harmful accumulation. However, in AD, APP is abnormally cleaved by β-secretase (BACE-1), followed by γ-secretase, leading to the formation of Aβ peptides, primarily Aβ40 and Aβ42. Among these, Aβ42 is more prone to aggregation, resulting in the formation of neurotoxic oligomers, which subsequently cluster into amyloid plaques within the CNS. These plaques contribute to synaptic dysfunction, neuroinflammation, and neuronal death, ultimately driving the progression of AD. An elevated Aβ42/Aβ40 ratio, together with increased total Aβ levels, is widely recognised as a key contributor to AD pathogenesis and is strongly linked to a heightened risk of developing the condition [34].

### Neurofibrillary Tangles (NFTs)

NFTs are composed of hyperphosphorylated tau protein aggregated into paired helical filaments [35]. The accumulation of tau disrupts the microtubule structure essential for axonal transport, thereby impairing intracellular trafficking and mitochondrial function. This impacts synaptic integrity and neuronal viability, playing a significant role in the neurodegenerative cascade characteristic of AD [36].

### Mitochondrial Dysfunction

Swerdlow, Burns, and Khan [37] suggest that mitochondrial dysfunction is a pivotal early event in the progression of AD. Impaired mitochondrial function results in reduced cellular energy production, which in turn initiates a cascade of neurodegenerative processes. Key consequences include the excessive generation of reactive oxygen species (ROS), disruption of calcium homeostasis, and dysfunction in mitochondrial quality control mechanisms such as mitophagy. Collectively, these alterations contribute to neuronal damage, synaptic loss, and ultimately, cognitive decline in AD [38].

### Oxidative Stress (OS)

Oxidative stress typically arises from the presence of free radicals and ROS, which are formed by molecules containing unpaired electrons from oxygen- and nitrogen-based compounds. In AD, the deposition of Aβ leads to the binding of these proteins to mitochondrial membranes, disrupting their function and prompting the production of ROS. This overabundance of ROS within neuron cell bodies exacerbates the condition, initiating processes such as elevated intracellular calcium levels, mitochondrial dysfunction, and DNA damage linked to oxidative stress [39].

## Gut–Brain Axis and AD

In AD, emerging research suggests that disruptions in the gut–brain axis may contribute to disease progression. The GBA is a bidirectional communication between the CNS and the enteric nervous system, maintaining a link between brain functions and peripheral intestinal functions. Alterations in gut microbiota composition and intestinal barrier integrity have been observed in individuals with AD. These changes may influence neuroinflammation, amyloid-beta accumulation, and synaptic dysfunction [40].

## Relationship Between Gut Microbiota and AD

In healthy humans, Bacillota (formerly Firmicutes) and Bacteroidetes make up over 90% of the gastrointestinal bacteria, alongside Actinobacteria, Proteobacteria, Fusobacteria, and Verrucomicrobia [41]. Alterations in these microbial communities have been documented in AD patients, with specific shifts observed in phyla such as an increase in Proteobacteria, Bacillota, and Bacteroidetes, and a decrease in Actinobacteria and Verrucomicrobia [42]. A reduction in Bifidobacterium (Actinobacteria) leads to a decrease in SCFAs such as butyrate, which are critical for maintaining gut barrier integrity and reducing inflammation [43]. Additionally, the decrease in *Akkermansia muciniphila* (Verrucomicrobia), a beneficial bacterium, compromises the gut barrier, increasing intestinal permeability and allowing lipopolysaccharides (LPS) to enter the bloodstream, triggering systemic inflammation and activating microglia in the brain [43]. The gut microbiota also appears to influence cerebrospinal fluid (CSF) biomarkers related to AD pathology [44]. Specific microbial families such as Clostridiaceae and Erysipelotrichaceae have been shown to correlate with reduced levels of CSF biomarkers like phosphorylated tau (p-tau), the Aβ42/Aβ40 ratio, and the p-tau/Aβ42 ratio [45]. Moreover, certain Bacillota species have been linked to decreased levels of amyloid-beta and tau proteins in both CSF and plasma, suggesting that these microbial communities may play a role in the regulation of AD-related proteins [46–48].

In contrast, the increased presence of *Alistipes* spp. and *Odoribacter splanchnicus* has been associated with higher levels of amyloid-beta in CSF and reduced levels of phosphorylated tau (p-tau) [48–50]. This suggests that these bacterial species might influence amyloid deposition in the brain, potentially by modulating gut–brain communication pathways. The mechanisms underlying these interactions are still being studied, but it is suggested that these bacteria may influence amyloid-beta accumulation through the production of bacterial metabolites that affect the immune system, gut barrier function, or brain inflammation [51].

In addition to these microbial changes, *Akkermansia muciniphila* has a complex relationship with neurodegenerative diseases like AD [52–54]. Some studies suggest that *A. muciniphila* can have beneficial effects, such as improving neurotransmitter production and enhancing the efficacy of SCFAs that support brain health. However, other research indicates that its reduction in the gut microbiota of AD patients may contribute to neuroinflammation and cognitive decline. This dual role suggests that *A. muciniphila*’s impact on AD progression may depend on the overall balance of the gut microbiota and the individual’s health status [55]. *A. muciniphila* may improve gut barrier integrity and promote the production of anti-inflammatory metabolites; however, its absence or reduced abundance may exacerbate neuroinflammation and contribute to the progression of AD [55]. Furthermore, *A. muciniphila* has been shown to influence short chain fatty acids (SCFAs) metabolism and neurotransmitter production, which have direct implications for the development and progression of AD [56–58]. SCFAs, particularly butyrate, are known for their neuroprotective effects, including the regulation of microglial activation, maintenance of blood–brain barrier integrity, and inhibition of neuroinflammation [59–61].

In AD, disruptions in SCFA production due to *A. muciniphila* depletion may contribute to microglial dysfunction and impaired cognitive function. On the other hand, the presence of *A. muciniphila* may help restore gut–brain communication, enhancing neurotransmitter balance and protecting against neurodegeneration. Therefore, the therapeutic potential of targeting *A. muciniphila* levels in the gut holds promise for future AD treatments that modulate gut microbiota composition to manage or slow disease progression. Consequently, alterations in *A. muciniphila* and SCFA levels may lead to changes in miRNA expression, which could further influence neuroinflammatory pathways and AD pathology [55]. Table 1 presents a summary of key gut microbiota changes influencing AD pathology, outlining the major bacterial shifts, their roles, and potential impact on disease progression.

**Table 1:** Key gut microbiota changes influencing AD pathology

Phylum/genus	Change in AD	Proposed role/mechanism	Impact on AD pathology	Reference
Bacillota	↑ Increased/altered composition	Produces SCFAs; altered levels influence inflammation and Blood brain barrier (BBB) integrity	May contribute to neuroinflammation and synaptic dysfunction	[49]
Bacteroidetes	↑ Increased/shifted ratio with Bacillota	Produces LPS; elevated levels linked to systemic inflammation	Promotes microglial activation and Aβ deposition	[42]
Proteobacteria	↑ Increased	Gram-negative bacteria produce LPS endotoxins	Triggers systemic inflammation and BBB breakdown	[50]
Actinobacteria	↓ Decreased	Includes Bifidobacteria, with anti-inflammatory functions	Loss may reduce neuroprotection	[48]
Verrucomicrobia	↓ Decreased	Includes Akkermansia muciniphila, maintains mucosal health	Reduction impairs gut barrier, increases endotoxin leakage	[42]
Lactobacillus spp.	↓ Decreased	Produces GABA and SCFAs; supports gut–brain axis	Decline linked to cognitive impairment	[44]
Bifidobacterium spp.	↓ Decreased	Anti-inflammatory, enhances gut integrity	Loss leads to gut permeability and inflammation	[48]
Escherichia/Shigella	↑ Increased	Pathogenic; produce pro-inflammatory endotoxins	Elevates systemic inflammation and Aβ pathology	[42]

## MicroRNA

MicroRNAs (miRNAs) are small, non-coding RNA molecules that play crucial roles in the regulation of gene expression. Despite their small size, miRNAs significantly influence various biological processes by targeting messenger RNA (mRNA) molecules for degradation or by inhibiting their translation into proteins. Through this post-transcriptional regulatory function, miRNAs modulate gene expression by repressing translation or promoting mRNA degradation, thereby influencing a wide array of cellular processes, including proliferation, differentiation, apoptosis, and metabolic homeostasis [62]. miRNAs are found in diverse organisms, including animals, plants, and even viruses, underscoring their evolutionary significance. In humans, dysregulation of miRNA expression has been implicated in various diseases, including cancer, cardiovascular disorders, neurodegenerative diseases, and immune-related conditions [62, 63].

In the context of AD, miRNAs have emerged as key molecular regulators involved in several pathological processes, including Aβ metabolism, tau phosphorylation, synaptic dysfunction, neuroinflammation, and neuronal apoptosis. Accumulating evidence suggests that dysregulation of specific brain and circulating miRNAs occurs early in the disease course, even before the onset of clinical symptoms. Several miRNAs such as miR-29, miR-146a, miR-132, and miR-155 are known to target genes involved in Aβ production (e.g. BACE1), inflammatory pathways (e.g. NF-κB signalling), and tau pathology. As such, miRNAs are not only considered potential mechanistic contributors to neurodegeneration, but they also hold promise as non-invasive biomarkers for early detection and progression monitoring due to their stability in biofluids like plasma, serum, and CSF [63].

### Significance of the Fecal MicroRNA in AD

Emerging evidence suggests that dysregulated fecal miRNAs are involved in key pathological processes in AD, including Aβ aggregation, tau hyperphosphorylation, neuroinflammation, and synaptic dysfunction (64–65). These miRNAs, derived from both host and gut microbiota, represent promising non-invasive biomarkers for early detection and monitoring of AD, with the added benefit of reflecting gut–brain axis disruptions. Recent investigations have revealed that specific fecal miRNAs are differentially expressed in AD, with distinct patterns of downregulation and upregulation (66–68). Downregulated miRNAs include miR-29a, which negatively regulates β-site amyloid precursor protein cleaving enzyme 1 (BACE1) involved in Aβ production, and miR-132, a key regulator of synaptic plasticity and neuronal survival (69). miR-128 has also been shown to be reduced in AD and may influence the abundance of *Akkermansia muciniphila*, a beneficial gut microbe, potentially exacerbating gut dysbiosis (70). In contrast, miR-106b, miR-146a, and miR-155 are found to be upregulated in AD and are involved in pro-inflammatory pathways and microglial activation (69). miR-223 has been linked to immune regulation through modulation of *Clostridium* species, while miR-9 contributes to epithelial integrity and has been associated with the regulation of *Bacteroides fragilis*, a bacterium implicated in AD-associated inflammation (71–73). These regulatory miRNAs may serve as key intermediaries in the gut–brain axis, impacting both microbial composition and host immune responses. miR-223, for example, enhances host defence by modulating neutrophil activation and reducing *Bacteroides* overgrowth, which has been linked to amyloid accumulation (71). Similarly, miR-128 reduces Enterobacteriaceae abundance and suppresses pro-inflammatory cytokines (70), while miR-9 helps maintain gut epithelial integrity, influencing the prevalence of SCFA-producing bacteria such as *Faecalibacterium* (73). Together, these miRNAs represent potential molecular links between microbial ecology and neurodegeneration. Further research is necessary to validate these candidate biomarkers across larger cohorts and to elucidate causal pathways through which fecal miRNAs modulate AD pathophysiology (74–75).

### Correlation Between miRNA Expression Changes and CSF Biomarkers

Alterations in CSF biomarkers, such as the Aβ42/Aβ40 ratio and phosphorylated tau (p-tau) levels, are closely linked to miRNA expression changes in AD. Reduced Aβ42/Aβ40 ratio correlates with increased levels of miR-125b and miR-146a, which drive neuroinflammation and impair Aβ clearance by downregulating neprilysin (NEP) and insulin-degrading enzyme (IDE) [76]. Similarly, elevated p-tau levels are associated with increased miR-132 downregulation, leading to disrupted synaptic plasticity and neuronal loss [77]. Inflammatory cytokines like IL-6 and TNF-α also influence miRNA expression, with high levels of these cytokines linked to upregulation of miR-155, further exacerbating neuroinflammation and tau pathology [78].

### Bidirectional Relationship Between Gut Microbiota and Host MicroRNA Expression

Emerging evidence supports a dynamic and bidirectional relationship between gut microbiota and host miRNA expression, with important implications for the pathogenesis and progression of AD. Gut microbes and their metabolites including SCFAs, secondary bile acids, and LPS can modulate host miRNA expression both locally in the gut epithelium and systemically within the CNS [79]. SCFAs such as butyrate, produced mainly by *Faecalibacterium prausnitzii*, *Roseburia *spp., and *Eubacterium *spp., upregulate neuroprotective miRNAs like miR-124 and miR-132, which support synaptic function and suppress neuroinflammation [79, 80]. Conversely, host-derived miRNAs can directly regulate microbial composition; miR-146a, miR-515-5p, and miR-1226-5p secreted into the intestinal lumen via exosomes can enter bacterial cells and alter gene expression, with miR-1226-5p promoting *Escherichia coli* growth and miR-515-5p suppressing *Fusobacterium nucleatum* [81].

Several miRNAs appear to mediate gut–brain interactions relevant to AD. miR-223 and miR-146a regulate intestinal barrier integrity and are induced by dysbiosis [82], while miR-155 and miR-21, often elevated in response to endotoxins from *Bacteroides fragilis* or Proteobacteria such as *Escherichia/Shigella*, are increased in both the gut and the AD brain [83, 84]. These miRNAs promote NF-κB activation, microglial reactivity, and cytokine release, contributing to early inflammatory stages of AD. SCFA-producing bacteria can also reduce the expression of pro-inflammatory miRNAs, whereas the overgrowth of pathobionts such as *Bilophila wadsworthia* and *Desulfovibrio *spp. may elevate miRNAs associated with barrier dysfunction and inflammation [85]. However, some findings challenge existing assumptions; for example, elevated butyrate levels have been reported in certain AD models despite concurrent microglial activation and cognitive decline, suggesting SCFAs may not always exert protective effects [86].

Despite these advances, important gaps and inconsistencies remain. miR-132 shows a robust decline in the AD brain, yet peripheral levels often remain unchanged, raising concerns about whether circulating miRNAs reliably reflect CNS pathology [87]. Similarly, *Akkermansia muciniphila* is linked to improved cognition in some cohorts but increased inflammation in others, pointing toward strain-specific or host-dependent effects [88]. Temporal mapping is also lacking; it remains unknown when microbiota–miRNA alterations arise across the AD continuum, as most studies are cross-sectional and cannot establish causality. Tissue-specific divergence presents another challenge, as it is unclear how miRNA signatures differ between gut, blood, and brain in response to microbial shifts, and which compartment best reflects AD-related pathophysiology. Additionally, sex-based differences in microbial composition and miRNA regulation are underexplored, despite their potential to explain cohort variability [65].

### MicroRNA Gene Expression and Cognitive Impairment in AD

Reduced abundances of gut microbes such as Rikenellaceae, unidentified Ruminococcaceae, and Alistipes, along with decreased microbial diversity, have been associated with higher risks of mild cognitive impairment (MCI) in AD [89]. These microbial shifts parallel reductions in key serum miRNAs, including hsa-let-7g-5p, hsa-miR-107, and hsa-miR-186-3p, which collectively suggest an early systemic link between gut dysbiosis and cognitive decline [86, 111]. Several fecal and circulating miRNAs show strong relevance to AD pathology. Reduced levels of miR-132, an essential regulator of synaptic plasticity and neuronal survival, have been reported in AD and may impair synaptic integrity, memory consolidation, and dendritic growth through dysregulation of CREB signalling [90, 91]. Experimental restoration of miR-132 has been shown to reduce tau hyperphosphorylation and neuronal degeneration, highlighting its therapeutic potential [92, 93]. miR-29a, which suppresses BACE1 and limits Aβ formation, is also downregulated in AD, supporting its role in amyloid accumulation and neurotoxicity [3, 4]. Meanwhile, dysregulation of other miRNAs, including miR-9, crucial for neurogenesis and neuronal connectivity, has been linked to disrupted synaptic function and cognitive processing [94, 95].

In contrast, several pro-inflammatory miRNAs are elevated in AD. miR-155 is consistently upregulated in fecal and peripheral samples and is strongly associated with microglial activation, cytokine release, neuroinflammation, and synaptic injury [93–97]. miR-146a and miR-106b, also increased in AD, contribute to inflammatory signalling and impaired Aβ clearance, partly by suppressing neprilysin and insulin-degrading enzyme [96, 98]. Regulatory miRNAs such as miR-223 and miR-128 additionally shape gut microbiota composition and gut barrier integrity by modulating species like Clostridium, Bacteroides fragilis, Enterobacteriaceae, and SCFA-producing bacteria, including Faecalibacterium [7–9]. Microbial metabolites—including short-chain fatty acids (SCFAs), secondary bile acids, and LPS—further influence host miRNA expression. SCFAs such as butyrate, produced by Faecalibacterium prausnitzii, Roseburia, and Eubacterium, enhance neuroprotective miRNAs like miR-124 and miR-132, whereas host miRNAs such as miR-1226-5p and miR-515-5p can directly regulate bacterial gene expression in species like *E. coli* and *Fusobacterium nucleatum* [4, 11]. Despite these insights, inconsistencies remain regarding SCFA levels, peripheral miRNA reliability, and species-specific microbial effects—underscoring the need for longitudinal, multi-tissue, and sex-stratified studies to clarify temporal dynamics and improve the diagnostic and therapeutic potential of fecal miRNAs in AD [5, 99–102].

### Mechanistic Pathways Linking miRNA Dysregulation to AD Pathology

A growing body of evidence supports the mechanistic role of miRNAs in driving core pathological processes in AD, reinforcing their biological plausibility. One of the most consistently altered miRNAs, miR-132, regulates synaptic plasticity, dendritic growth, and neuronal survival, and its loss disrupts the CREB signalling pathway, which is central to memory consolidation and long-term potentiation [3, 90, 91, 103]. Downregulation of miR-132 leads to impaired CREB-dependent transcription and reduced expression of synaptic proteins necessary for neuronal connectivity. Experimental restoration of miR-132 in AD models rescues synaptic function and reverses dendritic atrophy, demonstrating a direct causal link between miR-132 deficiency and synaptic degeneration [104–107].

Beyond synaptic dysfunction, miR-132 also influences tau phosphorylation and clearance, providing mechanistic insight into neurofibrillary tangle formation. Reduced miR-132 enhances the activity of tau kinases such as GSK-3β and CDK5, promoting tau hyperphosphorylation and aggregation [93, 94]. miR-132 deficiency further suppresses pathways responsible for tau degradation, including autophagy and proteasomal processing, thereby accelerating intracellular tau accumulation [8]. Studies show that reintroduction of miR-132 reduces phosphorylated tau and improves neuronal survival, emphasising its role in tau homeostasis [4, 5, 10, 11].

Multiple miRNAs, particularly miR-29a/b and miR-107, exert direct regulatory control over BACE1, the β-secretase responsible for amyloidogenic APP cleavage [3, 98]. Downregulation of miR-29 family members increases BACE1 expression, leading to excessive Aβ42 generation and early amyloid plaque deposition [107]. Similarly, reduced miR-107 expression correlates with increased BACE1 activity and accelerated disease progression, highlighting the role of miRNA-mediated β-secretase regulation in Aβ pathology [76, 77, 107]. Loss of miR-132 also indirectly enhances amyloidogenic processing, reinforcing its role as a central upstream regulator [104–107].

Pro-inflammatory miRNAs such as miR-155 and miR-146a further contribute to AD pathology through modulation of neuroimmune signaling. Upregulation of miR-155 amplifies NF-κB–mediated cytokine production by suppressing SOCS1, promoting a sustained inflammatory state in microglia [72–74, 117]. miR-146a, conversely, acts as a feedback regulator but becomes dysregulated in chronic inflammation, leading to abnormal suppression of IRAK1 and TRAF6 and altering innate immune responses in AD [78]. The presence of these inflammatory miRNAs in both gut and brain tissues links gut dysbiosis to central neuroinflammatory processes, demonstrating a mechanistic interface between peripheral and CNS pathology [79, 80].

## Impacts of Macronutrients and Amino Acids on miRNA Gene Expression

The interplay between macronutrients, amino acids, and miRNA expression plays a critical role in the progression and pathophysiology of AD. Macronutrients such as carbohydrates, proteins, and fats modulate miRNA expression, thereby influencing key molecular pathways implicated in AD, including insulin signalling, oxidative stress, neuroinflammation, and synaptic plasticity. Carbohydrates, especially glucose, impact miRNA profiles through mechanisms involving insulin resistance, a central feature of AD pathology. Dysregulation of glucose metabolism alters the expression of miRNAs such as miR-29, miR-146, and miR-34. Specifically, miR-29 downregulation in insulin-resistant states enhances BACE1 expression, leading to increased amyloid-beta production [108]. miR-146 is upregulated under chronic hyperglycaemia, suppressing inflammation regulators like IRAK1 and TRAF6, exacerbating neurodegeneration [109]. Overexpression of miR-34 further inhibits neuroprotective factors such as SIRT1, impairing synaptic plasticity and neuronal survival [110]. Proteins and specific amino acids also exert significant effects on miRNA expression. Leucine regulates miR-132, which is essential for dendritic growth and neuroprotection, by activating the mTOR pathway [111, 112]. Similarly, amino acids such as arginine and glutamine influence miRNAs associated with neuroinflammation and oxidative stress, contributing further to AD pathogenesis [113]. High-fat diets exhibit a dual effect: certain fat compositions worsen inflammation, while others, depending on the type of fat and duration of consumption, offer neuroprotective benefits by modulating miRNA expression [114, 115]. Dietary interventions aimed at modulating miRNA expression thus represent a promising therapeutic strategy for AD, albeit characterised by complexity and variability. Compounds such as omega-3 fatty acids, notably docosahexaenoic acid (DHA), enhance the expression of miR-132 and miR-124, promoting neuroprotection and synaptic integrity, while downregulating pro-inflammatory miRNAs like miR-146a [116]. Polyphenol-rich diets also influence miRNA expression beneficially; however, human studies show inconsistent findings, suggesting benefits may require sustained dietary intake [117]. Factors including genetic variation, gut microbiome composition, and disease stage contribute to variability in miRNA response to diet, complicating result interpretation [118]. The impact of high-fat diets on miRNA expression and neuroinflammation remains controversial. Diets rich in saturated fats are linked to upregulation of pro-inflammatory miRNAs such as miR-155 and miR-146a, implicated in neuroinflammation and cognitive decline. In contrast, ketogenic or high-fat, low-carbohydrate diets have shown potential to increase neuroprotective miRNAs like miR-124. These inconsistencies underscore the need for longitudinal and mechanistic studies to clarify causal relationships between diet and miRNA regulation in AD [119]. A deeper understanding of these interactions may support the development of precision nutrition strategies as adjunctive approaches to AD prevention and management, offering novel insights into targeting miRNA-mediated pathways through diet [120–122]. Table 2 outlines how specific nutrients influence microRNA expression, highlighting their roles in regulating key pathways such as inflammation, amyloid production, and synaptic function, all of which contribute to AD progression.

**Table 2:** Nutrient-linked miRNAs and their roles in AD pathophysiology

Nutrient/dietary component	Associated miRNA(s)	Regulatory pathway/target	Effect on AD pathology	Reference
Glucose/carbohydrates	miR-29	↓ miR-29 → ↑ BACE1	↑ Amyloid-β production	[108]
	miR-146	↑ miR-146 → ↓ IRAK1, TRAF6	↑ Neuroinflammation	[109]
	miR-34	↑ miR-34 → ↓ SIRT1	↓ Synaptic plasticity, ↑ degeneration	[110]
Leucine (amino acid)	miR-132	↑ miR-132 via mTOR pathway	↑ Dendritic growth, neuroprotection	[111, 112]
Arginine/glutamine	Various (e.g. miR-155)	Modulate oxidative stress and inflammation	↑ Neuroinflammation, oxidative damage	[113]
Saturated fat (high-fat diet)	miR-155, miR-146a	↑ Pro-inflammatory miRNAs	↑ Neuroinflammation, cognitive decline	[114]
Omega-3 fatty acids (DHA)	miR-132, miR-124	↑ Neuroprotective miRNAs	↑ Synaptic integrity, ↓ inflammation	[116]
	miR-146a	↓ miR-146a	↓ Pro-inflammatory response	[116]
Polyphenols	Various (e.g. miR-124)	Modulate inflammation and neuronal signaling	Mixed effects; potentially neuroprotective	[117]
Ketogenic diet (high-fat, low-carb)	miR-124	↑ miR-124	↑ Neuroprotection, ↓ inflammation	[119]

### Diet-Mediated Modulation of Gut Microbiota, miRNA Expression, and Cognitive Outcomes

Diet plays a central role in shaping gut microbial communities, and these microbial shifts directly influence host miRNA expression relevant to AD pathology [79]. Fibre-rich and anti-inflammatory diets promote the expansion of beneficial SCFA-producing taxa such as *Faecalibacterium prausnitzii*, *Roseburia *spp., and *Eubacterium *spp., which subsequently enhance the expression of neuroprotective miRNAs including miR-124 and miR-132 [79, 80]. Conversely, Western-style diets high in saturated fats and refined sugars promote dysbiosis characterised by increases in *Escherichia/Shigella*, *Bacteroides fragilis*, *Bilophila wadsworthia*, and *Desulfovibrio *spp., leading to elevated LPS and other inflammatory metabolites that upregulate miR-155, miR-21, and miR-146a [81–85]. These observations demonstrate that dietary patterns shape microbial ecology in ways that directly modulate the host miRNA environment [76–79].

Diet-induced microbial alterations also influence key miRNAs implicated in AD-related molecular pathways, including amyloidogenesis, tau phosphorylation, and synaptic plasticity [90, 91, 103]. SCFA-associated microbial profiles support the maintenance of miR-132 and miR-29a/b, which regulate BACE1 expression and CREB-mediated neuronal survival, thereby protecting against amyloid accumulation and synaptic loss [3, 98]. Dysbiosis caused by unhealthy dietary patterns suppresses these miRNAs, leading to increased BACE1 activity, enhanced Aβ production, and dysregulated tau kinase activity that accelerates neurofibrillary tangle development [104–107]. In parallel, inflammatory diets elevate miR-155 and miR-21, enhancing NF-κB activation and microglial reactivity, which amplify neuroinflammation and exacerbate cognitive decline [82–84, 97]. Thus, diet-driven microbial dysbiosis modulates miRNA networks that directly feed into established AD molecular mechanisms [90].

**Fig. 1:**
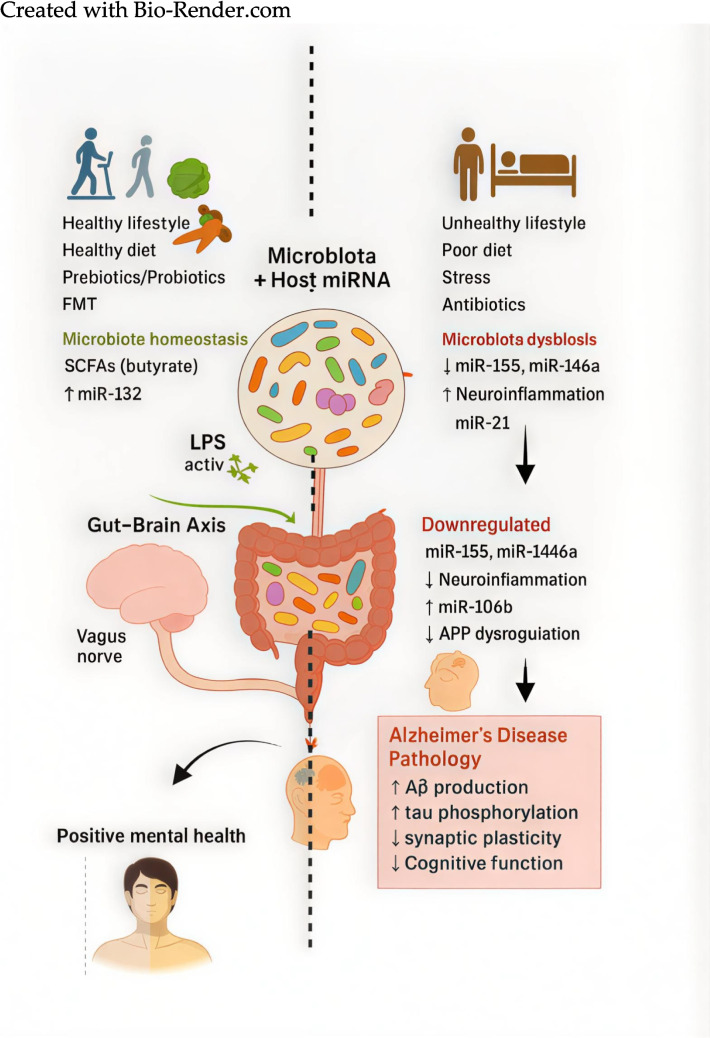
Interplay between diet, gut microbiota shifts, and microRNA regulation. Created with Bio-Render.com

Emerging evidence further links diet-mediated microbial and miRNA alterations to measurable cognitive outcomes across the AD spectrum [89]. Reductions in beneficial microbial taxa such as *Rikenellaceae*, *Ruminococcaceae*, and *Alistipes*, along with decreased circulating miRNAs including miR-107, miR-132, and hsa-let-7g-5p, correlate with a higher risk of mild cognitive impairment and early synaptic dysfunction [104–108]. Elevated fecal levels of miR-146a, miR-155, and miR-106b commonly associated with dysbiosis and pro-inflammatory dietary patterns are linked with increased gut permeability, systemic inflammation, and progression from MCI to dementia [93, 94]. miRNAs such as miR-223, miR-128, and miR-9, which are modulated by microbial shifts, also influence gut barrier integrity, immune activation, and neuronal connectivity, thereby contributing to cognitive deterioration [8, 98]. As illustrated in Fig. 1, dietary patterns influence gut microbial composition and microRNA-mediated regulatory pathways that collectively shape host–microbiome interactions.

### Unresolved Mechanistic Conflicts in miRNA Signatures and Microbial Interactions in AD

Despite increasing evidence supporting the role of gut microbiota and fecal miRNA in AD, several controversies and unresolved questions remain. A major debate concerns the dualistic effects of butyrate, a key SCFA. While butyrate is widely recognised for its neuroprotective actions, including anti-inflammatory effects, modulation of microglial activation, and upregulation of beneficial miRNAs such as miR-124 and miR-132 [59–61]. Other studies have reported paradoxical findings. In some AD models, SCFA levels, including butyrate, are elevated despite worsening pathology, suggesting that the impact of butyrate may be highly context dependent [86, 99]. These discrepancies indicate that butyrate’s effects may vary based on microbial composition, host metabolism, disease stage, and receptor sensitivity, highlighting the need for more mechanistic research to clarify whether SCFAs are uniformly protective or capable of contributing to pathology under specific conditions.

Another prominent controversy involves the strain-specific and host-dependent roles of Akkermansia muciniphila in neurodegeneration. Although A. muciniphila has been widely reported to enhance mucosal integrity, reduce inflammation, and support SCFA metabolism,mechanisms thought to be beneficial in AD [52–55].Other studies indicate contradictory outcomes. Some reports show that particular strains of A. muciniphila may exacerbate inflammation or metabolic stress depending on host factors [88, 100]. This suggests that its role in AD is complex, potentially beneficial in some physiological or dietary contexts while detrimental in others. These inconsistencies emphasise the importance of analysing A. muciniphila at the strain-level, rather than generalising its effects across all variants, and point toward a need for host–microbe interaction studies that incorporate immune status, diet, and microbial ecology.

A final unresolved issue concerns the discrepancies between fecal and brain miRNA profiles in AD. While fecal miRNAs provide a valuable window into gut–brain axis dysregulation, inflammatory states, and microbial shifts, their expression patterns do not always mirror those found in the CNS. For instance, miR-132 and miR-29a are consistently downregulated in AD brain tissue and are closely linked to synaptic failure, tau hyperphosphorylation, and impaired Aβ regulation [90–92, 98]. However, several studies report inconsistent, minimal, or unchanged levels of these same miRNAs in fecal or circulating samples [87, 99]. These discrepancies likely arise from tissue-specific regulation, differential release mechanisms, microbial interactions, and degradation differences between brain and stool environments. Additionally, technical factors such as RNA extraction variability, library preparation inconsistencies, and bioinformatic pipeline differences may further amplify these conflicting results [78, 79]. Overall, resolving these inconsistencies will require multi-tissue, longitudinal research directly comparing miRNA signatures from gut, circulation, and brain across preclinical, MCI, and dementia stages.

## miRNA-Based Therapeutic Approaches

Targeting miRNA dysregulation presents a promising therapeutic avenue for restoring gut microbiota balance and mitigating AD pathology. Synthetic miRNA mimics, such as miR-132 mimics, or inhibitors like anti-miR-146a, can specifically modulate miRNA levels to influence gut microbiome composition. These interventions reduce neuroinflammation, promote beneficial SCFA production, and support gut–brain homeostasis [122, 127]. Dietary interventions also naturally regulate miRNA levels, fostering microbial diversity. Polyphenol-rich foods (fruits, vegetables, tea) and omega-3 fatty acids (fish oils) positively influence miRNA expression, reducing inflammation linked to AD [127]. Bioactive compounds such as curcumin, resveratrol, sulforaphane, and epigallocatechin gallate (EGCG) contribute to modulating miRNA profiles involved in neuroinflammation, oxidative stress, and neuronal function. Fecal microbiota transplantation (FMT) emerges as a novel strategy to restore miRNA-microbiota interactions, potentially reversing cognitive decline in early AD stages by re-establishing microbial diversity and promoting beneficial miRNA regulation [128]. Preclinical and clinical trials explore miRNA-targeting strategies for AD. Nanoparticle-based delivery systems improve stability and brain targeting of miRNA mimics/inhibitors [129]. Animal studies show exosome-loaded miR-132 mimics rescue cognitive deficits and reduce tau pathology [130]. Early-phase clinical trials are assessing the safety and efficacy of miRNA-targeting oligonucleotides, including antisense oligonucleotides (ASOs), as disease-modifying agents [131].

### Personalised miRNA-Based Treatments

Personalised miRNA-based therapies tailored to individual gut microbiome profiles represent an emerging direction in precision medicine for AD, as both microbial composition and host miRNA signatures show strong inter-individual variability linked to cognitive outcomes [89, 90]. Integrating microbiome sequencing with miRNA profiling allows identification of patient-specific dysregulated pathways, such as reduced miR-132, miR-29a, and miR-107 or elevated miR-155, miR-146a, and miR-21, which directly influence amyloid processing, tau phosphorylation, neuroinflammation, and synaptic plasticity [3, 87, 98,]. This approach also enables targeted restoration of protective miRNAs that decline in AD such as miR-132, miR-124, and miR-128 or suppression of pathogenic ones, including miR-155 and miR-21, according to each patient’s molecular profile [91–93, 103]. Because gut microbial communities strongly shape host miRNA expression, incorporating individualised microbiome data may guide interventions aimed at modifying dysbiosis-associated miRNAs, especially those responsive to microbial metabolites such as SCFAs and LPS [79–82]. Precision strategies can further reduce off-target effects by aligning therapeutics with each patient’s unique miRNA–microbiota network, thereby improving safety and enhancing long-term disease modification potential [104–108].

### Translational Challenges in miRNA Therapeutics

Although microRNA-based therapies show strong preclinical promise in AD, their translation to humans remains limited by the difficulty of achieving efficient and targeted delivery across the BBB, even when using engineered miRNA mimics or inhibitors [104–107]. Off-target effects represent another major challenge because individual miRNAs regulate multiple gene networks simultaneously, increasing the likelihood of unintended alterations in synaptic, inflammatory, or metabolic signalling pathways [3, 98, 109]. Translational uncertainty is further increased by substantial differences between rodent models and human neurobiology, raising concerns about whether therapeutic strategies—such as restoring miR-132 can reliably replicate their beneficial effects when applied clinically in AD patients [104–107]. Additional complexity arises from the dynamic behaviour of disease-associated miRNAs, including miR-155, miR-146a, and miR-21, which shift in response to gut dysbiosis and systemic inflammation, making long-term therapeutic modulation in humans unpredictable [82–84, 97].

## Current Best Practices for Fecal miRNA Isolation, Enrichment, and Normalisation

Standardised pre-analytical handling is critical for reliable fecal miRNA profiling, as RNA in stool is highly susceptible to degradation and inhibition. Fresh samples should be collected into RNA-stabilising buffers or snap-frozen at − 80 °C as soon as possible, avoiding repeated freeze–thaw cycles that alter both microbial composition and miRNA integrity [4, 12]. Mechanical disruption (e.g. bead-beating) combined with chaotropic reagents improves lysis of host cells and bacteria but must be balanced against shearing of small RNAs and carry-over of PCR inhibitors such as bile salts and complex polysaccharides [4, 12, 79]. Comparative studies indicate that kits specifically optimised for low-abundance or stool-derived RNA, together with the inclusion of exogenous spike-in controls during extraction, provide more consistent recovery and permit correction for extraction efficiency across samples [4, 11, 12].

For downstream sequencing or array-based methods, best practice is to enrich for small RNAs and reduce background from rRNA, tRNA fragments, and long host transcripts. Size-selection steps (e.g. 18–30 nt gel or column purification) and optional rRNA depletion increase the proportion of informative reads mapping to miRNAs and reduce library complexity biases in next-generation sequencing (NGS) [4, 11, 12]. Quality control using fluorometric quantification and small-RNA electropherograms helps to identify degraded or inhibitor-contaminated samples before library preparation [4, 12]. Library construction protocols that minimise ligation bias, use unique molecular identifiers (UMIs), and apply sufficient PCR cycles to avoid over-amplification artefacts are recommended for fecal miRNA work, particularly when starting from low-input RNA [11, 123–126].

Normalisation remains a major source of variability in fecal miRNA studies, and current best practice combines biological and technical controls. Because there is no universally stable endogenous reference miRNA in stool, strategies often rely on global mean normalisation, small panels of empirically validated reference miRNAs, and/or exogenous synthetic spike-ins added at known concentrations [4, 12, 123–126]. Compositional data approaches and batch-effect correction (e.g. incorporating library size factors, RNA yield, haemoglobin or bacterial load proxies) further reduce technical noise and improve comparability across cohorts [123–126].

### MicroRNA Expression Profiling Techniques

A wide range of miRNA expression profiling technologies has been developed to support both research and clinical applications, with each platform offering distinct advantages depending on the biological question and sample type [123, 124]. These platforms differ in sensitivity, throughput, ability to detect novel miRNAs, and suitability for low-abundance or complex samples such as fecal material [123–126]. High-resolution methods such as next-generation sequencing (NGS) enable unbiased discovery and quantification of both known and novel miRNAs, including isomiRs and edited variants [123–125, 127–130]. In contrast, targeted platforms including qRT-PCR, microarrays, digital PCR (dPCR), and multiplex hybridisation assays provide high sensitivity for predefined miRNAs but lack the capacity to detect previously uncharacterised sequences [131–135]. Emerging spatial and single-cell sequencing approaches further enrich understanding of miRNA localisation and cellular heterogeneity, although these methods remain technically demanding and largely restricted to specialised research environments [136]. Table 3 provides a comparative overview of major miRNA detection platforms, highlighting key differences in analytical performance, including detection limits, dynamic range, and capacity for novel miRNA discovery.

**Table 3:** Comparative summary of miRNA detection platforms and their performance characteristics

Method/platform	Technical challenges	Key limitations	Suitability	Reference
NGS-miRNAs	Requires high-quality small RNA, complex library preparation and high sensitivity to inhibitors in fecal samples	Expensive, batch effects, ligation bias, and complex bioinformatics	Research**:** Excellent for discovery Clinical: Limited	[123–126]
qRT-PCR	Primer design critical, sensitive to inhibitors and requires reliable normalisation	Targets only known miRNAs and limited multiplexing	Research: Ideal for validation Clinical: High sensitivity;	[132]
Microarray analysis	Requires high input RNA; potential cross-hybridisation; lower precision in low-abundance miRNAs	Cannot detect novel miRNAs; narrower dynamic range than NGS	Research: Good for screening known miRNAs Clinical: Limited	[133]
Digital PCR (dPCR)	Requires clean, inhibitor-free RNA; high cost per reaction	Limited multiplex capacity; requires predefined targets	Clinical: Excellent for absolute quantification and biomarkers Research: Useful for low-abundance detection	[139]
Multiplex hybridisation assays	Requires high-quality RNA; dependent on probe design; moderate sensitivity	No discovery capability; lower sensitivity than PCR-based methods	Clinical: Suitable when RNA input is limited Research: Good for targeted panels	[135]
Single-cell miRNA sequencing	Technically complex; requires specialised instruments; very low RNA quantities	High cost; limited to research; challenging for stool-derived cells	Research: High-value mechanistic studies Clinical: Not yet feasible	[136]
In situ sequencing	Requires advanced imaging and tissue preparation; difficult optimization	Limited throughput; cannot analyse fecal samples directly	Research: Spatial localisation of miRNAs Clinical: Experimental only	[137]

## Current Knowledge Gaps and Future Directions

Although increasing evidence supports the relevance of gut microbiota–miRNA interactions in AD, significant knowledge gaps remain, particularly due to the scarcity of longitudinal studies capable of determining when shifts in fecal miRNAs and microbial composition emerge across the AD continuum [89, 90, 101]. Most existing studies are cross-sectional, making it difficult to disentangle cause–effect relationships between dysbiosis-associated miRNA changes such as reduced miR-132, miR-29a, and miR-107 or elevated miR-146a and miR-155 and subsequent cognitive decline [4, 7, 93, 94]. Another unresolved gap is the inconsistent alignment between fecal, circulating, and brain miRNA signatures, as shown by discrepancies in miR-132 and miR-29a levels across tissues, limiting their utility as reliable systemic biomarkers [87, 98, 104–107]. Additionally, variation in microbiome–miRNA interactions across sexes and populations remains understudied, despite evidence suggesting these factors may contribute to heterogeneity across AD cohorts [65, 101]. Further challenges arise from the lack of standardised methods for fecal miRNA extraction, sequencing, and normalisation, which restricts reproducibility and complicates comparisons across studies [4, 12, 79–82].

Future research should prioritize longitudinal fecal miRNA studies to identify early microbial and miRNA alterations that precede cognitive impairment, enabling clearer mapping of disease trajectories and mechanistic pathways in preclinical and prodromal AD [89, 90, 101]. Integrating multi-omics approaches including metagenomics, metabolomics, transcriptomics, immune profiling, and miRNA sequencing will be essential for uncovering how microbial metabolites such as SCFAs and LPS regulate key miRNAs including miR-124, miR-132, miR-155, and miR-21 [79–82, 92–94]. The development of harmonised, standardised protocols for fecal miRNA processing and bioinformatic analysis will improve cross-cohort comparability and facilitate large-scale discovery of robust biomarkers [4, 11, 12]. In addition, personalised therapeutic strategies informed by integrated miRNA–microbiota signatures may allow targeted restoration of protective miRNAs or suppression of pathogenic ones with greater precision and fewer off-target effects [104–108].

## Conclusion

Examining fecal miRNA profiles alongside dietary assessments offers a promising non-invasive method for diagnosing AD-related cognitive decline. This approach not only facilitates early detection but also allows for personalised treatment plans and interventions targeting dietary factors to reduce AD risk and slow progression. With the advancements in NGS, miRNAs have become valuable diagnostic tools in various diseases. However, there are significant gaps in our understanding of how fecal miRNA profiles relate to pathology related to AD, as research in this area has been limited. One notable gap lies in the comprehensive profiling of miRNAs in different stages of AD progression and in response to varying nutrient exposures. While numerous miRNAs have been implicated in AD pathology, current research often focuses on a subset of miRNAs, leaving many potential regulatory molecules unexplored. A deeper understanding of the complete miRNA profile in AD, particularly in relation to nutrient status, is essential for identifying novel biomarkers and therapeutic targets. This study aims to address these gaps by comprehensively analysing fecal miRNA profiles in relation to AD pathology, while also investigating potential associations with microbial metabolites such as SCFAs and examining the modulatory effects of macronutrients on miRNA gene expression.

## Supplementary Information

Below is the link to the electronic supplementary material.

ESM1 (DOCX 31.0 KB)

## Data Availability

No datasets were generated or analysed during the current study.
