# Supplementary material for: Unraveling the Significance of Fecal MicroRNA Profile in Alzheimer’s Disease
Source: Mol Neurobiol. 2025 Dec 22;63(1):319. doi: 10.1007/s12035-025-05626-6 (PMC12719353; doi:10.1007/s12035-025-05626-6)
Supplement: Supplementary file 1 — (DOCX 31.0 KB) [file 12035_2025_5626_MOESM1_ESM.docx]

# Supplementary table- fecal miRNAs reported in AD and their microbial associations and functional implications.

| **miRNA** | **Expression Change in AD** | **Microbial Associations** | **Functional Role in AD** | **Reference No.** |
| --- | --- | --- | --- | --- |
| miR-29a | Downregulated | Associated with AD-related dysbiosis patterns: ↓ Rikenellaceae, ↓ Ruminococcaceae, ↓ Alistipes; loss of SCFA-producers (Faecalibacterium, Roseburia, Eubacterium) | Regulates BACE1; ↓ miR-29a → ↑ Aβ production | [3,4,107] |
| miR-132 | Downregulated | Strongly linked to SCFA-producers: Faecalibacterium, Roseburia, Eubacterium | Synaptic plasticity, CREB signalling, neuronal survival | [84,91,92], [104–106] |
| miR-128 | Downregulated | ↓ Enterobacteriaceae, ↓ Akkermansia muciniphila | Anti-inflammatory; maintains metabolic gut balance | [7,9,70] |
| miR-9 | Altered | Regulates Faecalibacterium; Bacteroides fragilis | Neurogenesis, synaptic connectivity | [71–73], [94,107] |
| miR-106b | Upregulated | Associated with inflammatory taxa dominance: Proteobacteria, Bacteroides fragilis, Escherichia/Shigella | Inflammation, microglial activation | [69,96] |
| miR-146a | Upregulated | Induced by Bacteroides fragilis, Proteobacteria, Escherichia/Shigella | IRAK1/TRAF6 signalling; reduced Aβ clearance | [76],[83-84] |
| miR-155 | Upregulated | Triggered by endotoxins from Bacteroides fragilis, Escherichia/Shigella; linked to Proteobacteria expansion | Pro-inflammatory; microglial activation | [66–68], [83, 84 ,93 ,117] |
| miR-223 | Altered | Regulates Clostridium spp.; reduces Bacteroides overgrowth | Gut barrier protection; immune modulation | [71–73], [82], |
| miR-124 | Upregulated (SCFA-driven) | Upregulated by Faecalibacterium, Roseburia, Eubacterium | Neuroprotective; reduces inflammation | [4],[79–80], |
| miR-1226-5p | Regulatory | Promotes growth of Escherichia coli | Host → microbe gene regulation | [11,81] |
| miR-515-5p | Regulatory | Suppresses Fusobacterium nucleatum | Shapes gut community structure | [11,81] |
| miR-125b | Upregulated | Linked to AD-related dysbiosis: ↓ SCFA-producers; ↑ inflammatory taxa (B. fragilis, Proteobacteria) | ↓ NEP/IDE → impaired Aβ clearance | [76,98] |
